# Genomic population structure and insecticide resistance mechanisms in the malaria vector An. coluzzii across contrasting bioclimatic zones in West Africa

**DOI:** 10.21203/rs.3.rs-7878288/v1

**Published:** 2025-11-05

**Authors:** Enock K. Amoako, Kelly L. Bennett, Anastasia Hernandez-Koutoucheva, Isaiah Debrah, Collins M. Morang’a, Stephen Binaansim, Victor A. Asoala, Cristina Ariana, Keziah L. Malm, Gordon Awandare, Alistair Miles, Chris S. Clarkson, Lucas N. Amenga-Etego

**Affiliations:** West African Centre for Cell Biology of Infectious Pathogens (WACCBIP), University of Ghana; Genomic Surveillance Unit, Wellcome Sanger Institute, Hinxton; Genomic Surveillance Unit, Wellcome Sanger Institute, Hinxton; West African Centre for Cell Biology of Infectious Pathogens (WACCBIP), University of Ghana; West African Centre for Cell Biology of Infectious Pathogens (WACCBIP), University of Ghana; West African Centre for Cell Biology of Infectious Pathogens (WACCBIP), University of Ghana; Navrongo Health Research Centre, Ghana Health Service; Genomic Surveillance Unit, Wellcome Sanger Institute, Hinxton; National Malaria Elimination Programme, Ministry of Health; West African Centre for Cell Biology of Infectious Pathogens (WACCBIP), University of Ghana; Genomic Surveillance Unit, Wellcome Sanger Institute, Hinxton; Genomic Surveillance Unit, Wellcome Sanger Institute, Hinxton; West African Centre for Cell Biology of Infectious Pathogens (WACCBIP), University of Ghana

## Abstract

Environmental barriers influencing the movement of insect vectors can govern adaptive gene flow, including the dispersal of insecticide resistance mechanisms that compromise population control. We sought to understand population connectivity of the major malaria vector, *An. coluzzii*, across the different bioclimatic zones of West Africa using SNPs from whole genomes and inversion karyotypes previously associated with environmental adaptation. We identified restricted gene flow between populations from northern savannah and southern forested regions. Using Ghana as a case study, we found marked differences in insecticide resistance profiles across the different bioclimatic zones suggesting that population connectivity impacts on adaptive allele sharing. Greater evidence for target site pyrethroid and metabolic cross-resistance in the North reflects differences in insecticide use across the country. We also observed distinct resistance mechanisms in the coastal region of Greater Accra which may result from intense urban agricultural activity. Overall, findings suggest that environmental conditions restrict *An. coluzzii* gene flow to impact the geographical distribution of molecular insecticide resistance.

## Introduction

Insect vectors with wide geographical distributions are subject to varying selection pressures driven by diverse ecological conditions. Both geographical barriers and ecological selection on insect vectors can promote variation in physiological and behavioral traits that impact human disease transmission. For example, transmission can be impacted by differences in geographical species distribution, blood-feeding behavior, dispersal and habitat use[1]. Ecological divergence often impacts on gene flow to form genetically differentiated ecotypes or species with different abilities to act as disease vectors e.g., tsetse flies that are vectors for sleeping sickness[2] or mosquitoes that transmit malaria[3]. Differences in population connectivity underpinned by vector ecology also impacts the sharing of adaptive alleles across geographical space. For example, these could be adaptations which promote behavioral avoidance or resistance mechanisms, such as insecticide target site or metabolic resistance, which compromise the population control strategies public health relies on[4]. There is therefore a need to understand how geography, ecology and anthropogenetic selection pressures, such as the application of insecticides, interact to influence the effectiveness of disease reduction strategies.

Ghana, with its rich tapestry of ecological landscapes ranging from coastal Savannah, through dense tropical rainforests, to Sahelian Savannah is representative of the north to south environmental gradient that spans the countries of central West Africa. Its diverse ecological conditions impact the distribution of disease vectors, including major malaria vectors in the *Anopheles* mosquito genus[5]. Malaria burden and seasonal peaks in incidence vary markedly across Ghana resulting in ~ 500,000 infections a year[6]. The key malaria vectors in central West Africa include members of the *Anopheles gambiae s.l*. complex and *Anopheles funestus s.l* species group. Within the former is the major malaria vector *An. coluzzii*. Due to its ecological plasticity, it has a widespread distribution across Ghana including in the northern Savannah where the malaria burden is highest[7, 8]. It also exhibits considerable variation in physiological and behavioral traits across the different bioclimatic zones of the West African subregion. These include differences in mating behaviour[9], dispersal strategies[10] and phenotypes promoting thermal tolerance including entering into a state of dormancy during dry conditions, i.e., aestivation[11]. *An. coluzzii* also showcases genomic variability across contrasting environmental conditions. For example, a striking cline in 2La and 2Rb inversion frequencies across the bioclimatic zones of West and Central Africa have been observed in Nigeria, Burkina Faso and Cameroon[12–15]. While populations in arid, savanna regions exhibit higher frequencies of the inverted 2La and 2Rb karyotypes, those in humid, forested areas show lower frequencies[16, 17]. Experimental work has also associated these inversion karyotypes with feeding behavior[18] and thermal tolerance[19]. Furthermore, these karyotypes can impact resistance to *Plasmodium* parasite infection[20] and to insecticides used for population control[21, 22], highlighting that environmental adaptation can impact directly on factors influencing malaria transmission.

A major hurdle to malaria control is the rapid evolution of insecticide resistance in vector populations[23] which undermines the effectiveness of control tools including insecticide-treated nets (ITNs) and indoor residual spraying (IRS)[24, 25]. Resistance mechanisms include target site resistance and metabolic resistance (e.g., heightened activity of detoxification enzymes). Target site resistance is conferred by non-synonymous substitutions in the voltage-gated sodium channel (*Vgsc*) gene[26, 27], coding for the protein targeted by widely-used pyrethroid insecticides, the acetylcholinesterase (*Ace-1*) gene[28, 29] which codes for the protein target of carbamates and organophosphates, and the GABA receptor subunit *Rdl* gene, coding for the target of dieldrin[30, 31]. Metabolic resistance is provided by increased activity of enzyme products determined by key gene classes such as cytochrome P450 monooxygenases (P450s)[32, 33], esterases including carboxylesterases (Coe)[34–36], and glutathione S-transferases (Gst) [36]and uridine diphosphate (UDP)-glycosyltransferases[37]. Research conducted in Burkina Faso and Ghana has revealed that *An. coluzzii* populations in urban areas often exhibit higher levels of insecticide resistance phenotypes compared to rural populations[38, 39]. The urban/rural disparity has been attributed to differential exposure to insecticides used in agriculture and public health in peri-urban and urban settings. The distribution of insecticide resistance mechanisms is therefore expected to vary across geographical space and with environmental characteristics. Moreover, resistance loci like target-site substitutions and copy number variants at metabolic resistance loci vary geographically, often reflecting localized selection pressures[40–42]. Furthermore, scans for signals of recent selection have highlighted potential differences in insecticide resistance loci including *Vgsc* and cytochrome P450’s between forest and non-forest ecosystems in southern Ghana, suggesting differential selection pressures in the contrasting bioclimates[43]. Understanding the geographic distribution of these resistance mechanisms and how environmental factors influence the sharing of adaptive alleles is crucial for designing targeted and effective control strategies.

Advances in genomics have revolutionized our ability to investigate population structure and molecular insecticide resistance mechanisms in malaria vectors at high resolution[44–46]. Here we use whole-genome sequencing of samples collected in Ghana, alongside data available in the MalariaGEN Vector Observatory (https://www.malariagen.net/vobs/), to elucidate population structure across the different bioclimatic zones of West Africa and investigate if ecological variation promotes population structure in *An. coluzzii*. Using SNP, haplotype and CNV variation data, we also explore how molecular insecticide resistance varies across geographical space using Ghana as a case study to determine if connected populations are more likely to share adaptive alleles resulting from the selection pressures imposed by front-line public health.

## Methods

### Mosquito sampling and identification

Adult wild caught mosquitoes were sampled across Ghana from different ecological zones in cross-sectional studies from 2016 to 2018. Mosquito sampling was done in Navrongo, Upper East Region of the northern Savannah, Adansi in the Ashanti Region and Koforidua in the Eastern Region of the middle transitional forest, Madina in Greater Accra and Takoradi in Western Region of the coastal Savannah of Ghana. *Anopheline* mosquitoes were caught using Human Landing Catches (HLCs) at night, CDC light traps hanged overnight, and pyrethroid spray collections of resting mosquitoes in the early mornings. Adult female *An. gambiae s.l* were identified using morphological keys[47] and stored singly in 70% ethanol in 96 well PCR plates. These were then shipped to the Wellcome Sanger Institute Genomic Surveillance Unit. DNA was extracted using the Qiagen DNeasy blood and Tissue Kit (Qiagen Science, MD, USA) according to the manufacturer’s instructions before sequencing and genotyping. Genomic data from other West African countries were openly available through the Vector Observatory (https://www.malariagen.net/vobs/), a collaborative project to obtain genomics data of *Anopheles* mosquitoes for malaria control.

#### Sequencing and variant calling

Using previously described protocols described by the *Anopheles gambiae* 1000 Genomes (Ag1000G) phase 3 project (The Anopheles gambiae 1000 Genomes Consortium 2018, 2020), Illumina paired-end sequencing was performed with HiSeq 2000 and HiSeq X technologies. Briefly, 100–150bp sequencing reads were aligned to the AgamP4 PEST reference genome using BWA version 0.7.15[48] and single nucleotide polymorphisms (SNP) called using GATK version 3.7.0[49]. The resulting data was quality controlled to only include individuals with ≥ 10X overall median coverage and with data across greater than 50% of the reference genome. Samples identified as cross-contaminated by a percentage of ≥ 4.5% were excluded as defined by Ag1000G phase 3 project protocols. Sites where SNP calling and genotyping were expected to be unreliable based on previous analyses of Mendelian inheritance in laboratory crosses were also excluded from analysis.

Haplotypes were phased using a WDL implementation of read-backed phasing using WhatsHap v1.0[50] and statistical phasing using SHAPEIT v4.2.1[51] (https://github.com/malariagen/pipelines). Copy number variants (CNVs) for each individual were called based on the copy number state inferred across windows of the genome. The copy number state was normalised using a Gaussian hidden Markov model (HMM) implemented by hmmlearn (https://github.com/hmmlearn/hmmlearn) as described previously[52]. A CNV was called across regions where at least five contiguous genomic windows had a predicted copy number > 2 or > 1 for the X chromosome in males. To increase reliability, only CNV calls with a high likelihood > 1000 and low coverage variance < 0.35 based on the HMM were retained. All preceding analyses were performed with the malariagen_data python package.

#### Taxonomic status

Taxa were provisionally assigned to the samples using Ancestry Informative Markers (AIMS). These are a set of SNPs previously described by the *Anopheles gambiae* 1000 Genomes project as exclusive to each taxonomic group based on data generated from the *Anopheles* 16 genomes project[53]. A set of 2612 markers were used to differentiate sister species *An. gambiae*/*An. coluzzii* from the more divergent *An. arabiensis*, identified when the fraction of arabiensis-like alleles was > 0.6. A set of 700 AIMs were used to differentiate *An. gambiae* from *An. coluzzii* with samples scored as *An. gambiae* when the fraction of coluzzii-like calls was < 0.12 and *An. coluzzii* where this fraction was > 0.9. Individuals in-between these fractions represent other taxa. The taxonomic status of individuals was then confirmed with both PCA and an unrooted Neighbour-joining tree using 100,000 biallelic SNPs evenly spread across chromosome three because this region is unaffected by structural rearrangements such as inversions. Chosen SNPs had a minor allele frequency greater than 0.2% and no missing data. The Neighbour-Joining tree was constructed using city block distance.

#### Population structure

To investigate population structure, Principal Component Analysis (PCA) dimensionality reduction and Neighbour-joining trees were constructed for *An. coluzzii* using the 3L chromosome as applied for taxonomic analysis. We also performed PCA focused on the allele counts at two inverted regions of the genome previously shown to segregate among *An. coluzzii* from different environments[16, 54]. These regions are located on the 2L (2L:20,528,089 − 42,165,182) and 2R (2R:19,444,433 − 26,313,071) chromosomes. Inversion karyotype frequencies within each population cohort were assessed by typing individuals for inversion status using correlated tag SNPs as previously described[55]. We also assessed genomic differentiation among population cohorts using Hudson’s pairwise F_ST_ using the 3L chromosome arm[56]. To explore the genetic diversity and demography of populations, informative summary statistics were then calculated including Nucleotide diversity (θπ), Watterson’s theta (*θ*_*W*_) and Tajima’s D using cohorts with the malariagen_data python package. Statistics were only calculated for population cohorts with a minimum of ten individuals.

#### Insecticide resistance

To investigate the presence of known target site mutations associated with insecticide resistance we calculated amino acid substitution frequencies at genomic sites of interest for each population cohort. These were based on the occurrence of non-synonymous SNPs at an appreciable frequency present at greater than 5%. Regions included the voltage-gated sodium channel (Vgsc; AGAP004707) as the target gene of pyrethroids, the glutathione S-transferase gene which confers resistance to DDT (*Gste2*; AGAP009194), the Resistance to dieldrin gene (*Rdl*; AGAP006028) and the organophosphate target gene, acetylcholinesterase (*Ace1*; AGAP001356). We also calculated the frequencies of copy number variants (CNVs) for genes associated with insecticide resistance and present at greater than 5% frequency. These included the Cytochrome P450 gene’s (AGAP002862-AGAP002870, AGAP000818, AGAP008212-AGAP008219), carboxylesterases (AGAP006228, AGAP006723-AGAP006728), *Ace1* (AGAP001356) and *Gste2* (AGAP009194).

### Selection scans

To identify novel regions of the genome under selection we utilised the H12 homozygosity statistic across windows of the genome[57] with statistical peaks representing either a hard or soft sweep. The statistic was calculated using an optimal size of 1500 windows. This was identified by plotting the distribution of H12 values across different window sizes and identifying when values fell below 0.1 for the 95th percentile.

#### Diplotype clustering

To investigate which variants are associated with clusters of diplotypes under selection (i.e., regions of diploid genotypes), hierarchical clustering was performed using city block genetic distance and complete linkage using the malariagen_python package[34]. Observed amino acid substitutions and CNV variants were plotted onto the resulting dendrogram. Variants uniquely associated with clusters of diplotypes with low heterozygosity are candidates under selection.

## Results

### Population Sampling

**Figure 1:**
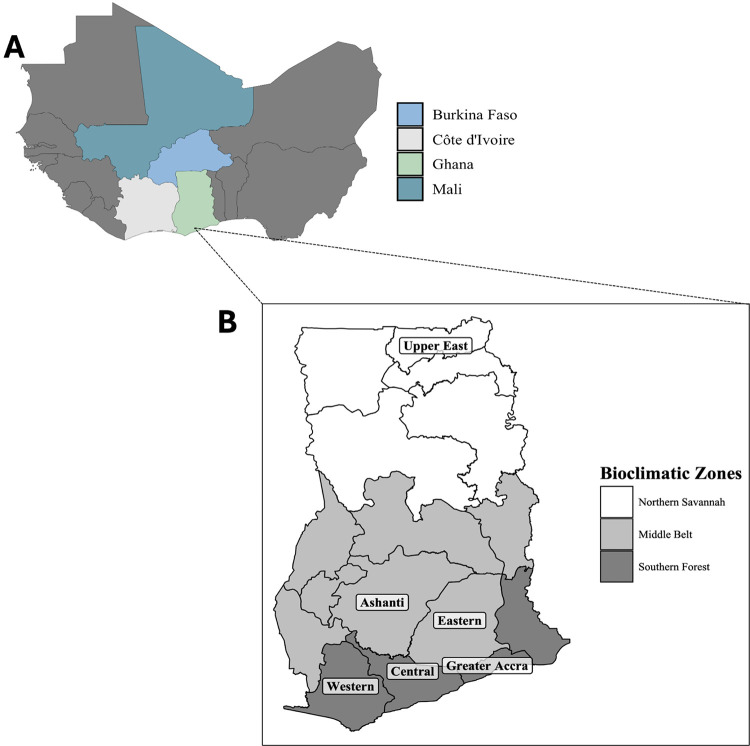
**A:** Map of West Africa highlighting countries included in the analysis including Ghana (green), Burkina Faso (light blue), Côte d’Ivoire (gray), and Mali (dark blue). **B:**Map of Ghana and its bioclimatic zones including the Northern Savannah (white), Middle Belt (light gray), and Southern Forest (dark gray).

*Anopheles* gambiae *sensu lato* samples were collected from 2016 to 2018 across the arid savannah region of the Upper East in northern Ghana. Of 1663 samples sent for whole genome sequencing, 1473 individuals passed quality controls as defined by the *Anopheles gambiae* 1000 Genomes (AG1000G) project[44, 58]. From these samples, 1324 mosquitoes were identified as *An. coluzzii* through PCA and AIMs analysis generated during data curation[44, 58] (Table 1). In addition, we included 486 *An. coluzzii* genomes from a previous study in Ghana[40] (Table 1). The sample sets included mosquitoes collected from 2012 to 2018 across five administrative districts located in the wet humid and deciduous forest region of South Ghana (Fig. 1,, Fig. S1, Supplementary Table 1). For comparison across the West African region, we used data made publicly available in the AG1000G Phase 3 resource (https://malariagen.github.io/vector-data/ag3/ag3.0.html). The sample sets included *An. coluzzii* collected from 2004 to 2014, originating from the dry savannah regions of Burkina Faso (n = 135) and Mali (n = 90) or from the wet humid region of southern Cote d’Ivoire (n = 80) (Fig. 1, Fig. S1). Sample sets were classified for analysis as a population cohort based on the administrative district and year of collection. Samples sequenced during this study had a median coverage of 35X which generated 162,714,957 SNPs on alignment to the AgamP3 genome. Of these SNPs, 52,946,551 were biallelic and segregated within the samples.

#### Geographical population Structure of An. coluzzii in Ghana

**Figure 2:**
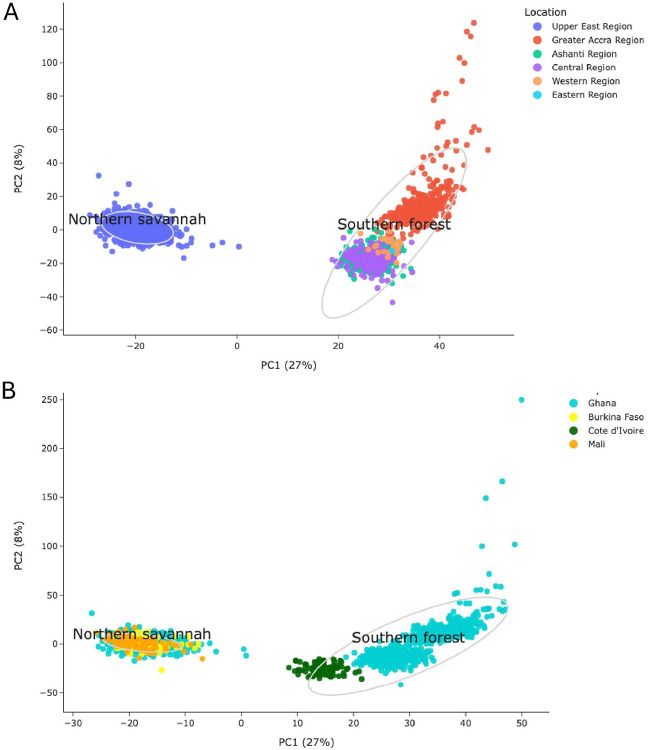
Plotting from Principal Components Analysis to investigate population structure in *An. coluzzii* collections from **A.** Ghana and **B**. from Ghana and the surrounding West African countries of Burkina Faso, Cote d’Ivoire and Mali using 10,000 SNPs on chromosome 3L. Grey circles represent 95% confidence centroid ellipses of two K-means clusters.

**Figure 3:**
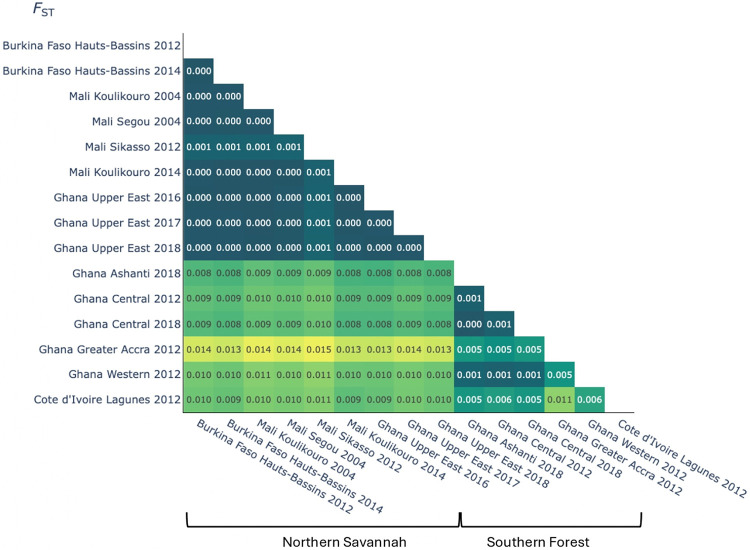
Hudson’s pairwise F_ST_ between population cohorts from Ghana and nearby West and Central African countries including Burkina Faso, Mali and Cote d’Ivoire. Lighter shades of green indicate higher values of F_ST_.

Population connectivity and consequent gene flow can influence the spread of adaptive alleles associated with insecticide resistance or vector competence for disease transmission. To first assess population connectivity of *An. coluzzii* across the different bioclimatic zones of Ghana, we implemented both principal components analysis (PCA) and neighbor-joining trees (NJT) using 100,000 single nucleotide polymorphisms (SNPs) spanning the 3L chromosome region (3L:15,000,000–41,000,000), which is free from structural rearrangements that could bias inference. Both the principal component analysis (PCA) and neighbor-joining tree (NJT) presented two major clusters of *An. coluzzii* (Fig. 2A and Fig. S2). One cluster included samples from the northern arid savannah region of the Upper East admin region only. The second included all populations from southern Ghana including the wet and humid deciduous forest regions of Greater Accra, Ashanti, Central, Eastern and Western Ghana. Although some southern comparisons were from a different collection time to the northern cohorts, two southern cohorts from Ashanti and the Central region were sampled in the same year. Furthermore, since an older 2012 cohort from the Central region clusters with a later cohort from the same region in 2019, we can observe that the pattern of population structure between the north and south of Ghana has held across time. Genetic differentiation was also lower within northern and southern Ghana cohorts (F_ST_ 0.000–0.005) but higher between these regions (F_ST_ 0.008–0.013), suggesting restricted gene flow between the north and south of the country (Fig. 3). Additionally, we observed that *An. coluzzii* from Greater Accra from 2012 diverged from the main cluster of individuals from southern Ghana on the PCA plot and appeared as a cluster of particularly closely related individuals in the Neighbor-Joining tree. This comparison included southern cohorts collected from the same time point. F_ST_ was also higher when comparing Greater Accra with the other southern population cohorts (F_ST_ 0.005). Although nucleotide diversity values did not differ substantially between any population cohort (Fig. S3), the Greater Accra cohort had a slightly higher Tajima’s D indicating it may have experienced greater genetic drift. Overall findings suggest high connectivity among southern populations but restricted gene flow between the different bioclimatic regions of northern and southern Ghana.

To investigate further whether population structure in *An. coluzzii* could be associated with different bioclimatic zones more widely across West Africa, we extended our analysis to include the West African populations of Burkina Faso, Cote d’Ivoire and Mali available through the Vector Observatory (https://www.malariagen.net/vobs/). Once again, we observed two major PCA and NJT clusters (Fig. 2B and Fig. S4). One included northern Ghana and the arid savannah regions of Mali and Burkina Faso. Another included southern Ghana along with southern Cote d’Ivoire, which both experience similar wet humid climate conditions. In support of these findings, F_ST_ was lower between the northern cohorts from Ghana, Burkina Faso and Mali (F_ST_ 0.000–0.001) and between southern Ghana and Cote d’Ivoire (F_ST_ 0.005–0.006) (Fig. 3). In contrast, F_ST_ was higher between northern and southern comparisons (F_ST_ 0.008 − 0.0015). These results support the notion that gene flow between *An. coluzzii* is restricted between populations found in the arid savannah and wet humid forest environments of West Africa.

**Figure 4:**
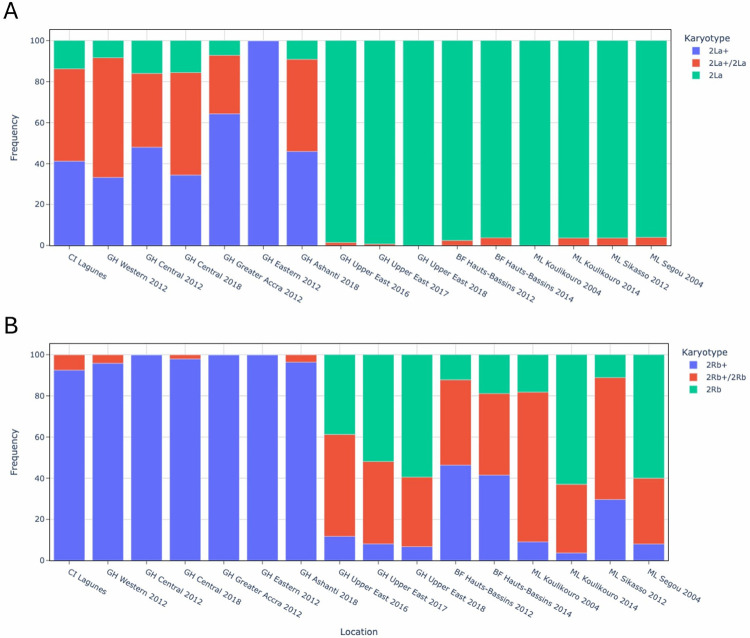
The frequency of karyotypes of the A. 2La and B. 2Rb inversion region in *An. coluzzii* from Ghana (GH) and the surrounding West African countries of Burkina Faso (BF), Mali (ML), and Cote d’Ivoire (CI). Southern forest locations include the Western, Central, Greater Accra, Eastern and Ashanti regions in Ghana and Lagunes in Cote d’Ivoire. Northern Savannah locations include the Upper East region in Ghana and locations in Burkina Faso, Mali, and Cote d’Ivoire.

To further investigate gene flow among *An. coluzzii* in west and central Africa, we investigated two inverted regions of chromosome two previously associated with different climate conditions[54]. We used tagging SNPs, correlated with inversion status, to assess the frequency of the different inversion karyotypes in each population cohort[19]. One major 2La inversion karyotype was shared between *An. coluzzii* from the northern arid regions of Ghana, Burkina Faso and Mali, suggesting unrestricted gene flow among these population cohorts (Fig. 4A). The inverted 2La inversion prevalent at 96–100% frequency in the northern populations has been associated with arid environments and thermotolerance[59, 60] (Table 2). In contrast, inverted 2La was between 0–16% frequency in the southern cohorts while the standard 2La + karyotype associated with mesic environments[54] was dominant at 33–100% frequency. Analysis of the 2Rb inversion (2R:19,444,433 − 26,313,071) presented similar findings in that the southern and northern populations had different dominant inversion karyotypes (Fig. 4B). However, findings differed in that all three 2Rb karyotypes were present in the northern regions while one form prevailed in the southern populations. Frequency analysis based on inversion tagging SNPs revealed that the dominant southern karyotype was the standard 2Rb + chromosomal form present at 93–100% (Table 2). Although all three karyotypes were present in the northern populations, the inverted 2Rb karyotype and heterozygote form reached higher frequencies (11–63% and 32–73%, respectively) than standard 2Rb+ (4–46%) and were more prevalent on average in the north (37% and 45% for 2La and the heterozygote, respectively) than the south (0% and 4%, respectively). Findings are consistent with the observation that inverted 2Rb generally appears at higher frequencies in arid environments while standard 2Rb + is associated with mesic environments[54] and supports restricted gene flow between the northern Sahelian and southern forest regions of West Africa characterised by different bioclimatic zones.

### Resistance to Insecticides

**Figure 5:**
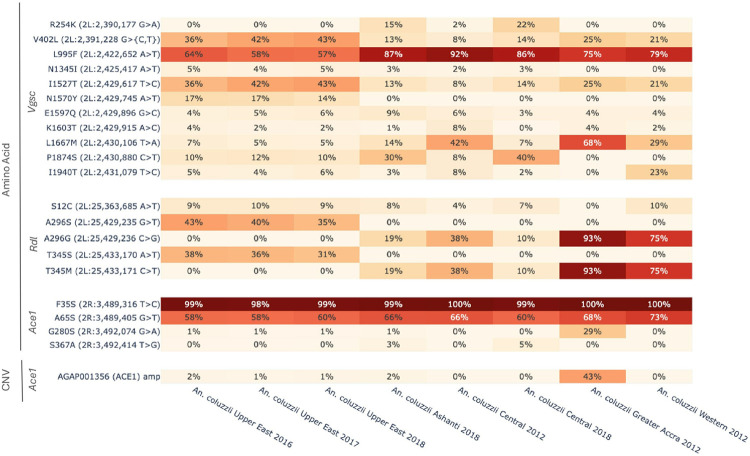
Amino acid and CNV frequencies at target site insecticide resistance loci including *Vgsc*, *Rdl* and *Ace1* in population cohorts from Ghana. Darker shades of red indicate higher frequencies.

To investigate geographical differences in insecticide resistance, we focused our analysis on the *An. coluzzii* data from Ghana in West Africa, which included country-wide sampling and the most recent timepoints for comparison. First, substitutions associated with target-site were investigated. Amino acid allele frequencies of three genes that encode for insecticide binding targets were computed: *Vgsc* (AGAP004707), *Rdl* (AGAP006028), and *Ace1* (AGAP001356). The *kdr* allele *Vgsc-L995F* established as conferring insecticide resistance to pyrethroids[27, 58] was identified in all populations but differed between the northern and southern populations with frequencies ranging from 57–64% and 86–92%, respectively (Fig. 5). Similar frequencies of Vgsc-*L995F* within the latter range were observed in the southern cohorts across the different years of collection ranging from 2012 to 2018. Additionally, we observed a double substitution, *Vgsc-V402L* and *I1527T*, at 36–43% frequency in northern Ghana and 8–25% in the south, including directly comparable cohorts sampled from the different locations in 2018. The double substitution previously observed in *An. coluzzii* from Burkina Faso and Kenya[42, 61] provides pyrethroid and DDT resistance at a reduced fitness cost compared to *L995F*[62]. As a result, the substitution pair is expected to replace *L995F* as the dominant *Vgsc* insecticide resistance mechanism. Similar to a study from Burkina Faso[61], we observed the *V402L* and *I1527T* substitution pair increasing in frequency. Frequencies increased 7% from 2016 to 2018 in northern Ghana while the frequency of *L995F* reduced over the same period, advocating for a shift in the main *Vgsc* resistance allele in West Africa.

We also observed both the allele pairs *A296G*/*T345M and A296S*/*T345S* at the *Rdl* locus, which are known to confer resistance to organochlorines such as dieldrin[63, 64] (Fig. 5). However, we found a difference in their geographical distribution. We observed *A296G* and *T345M* in all cohorts from southern Ghana only, while *A296S* and *T345S* were only found in northern Ghana. This provides support for our finding that gene flow is restricted across Ghana, including at the 2La inversion region on which *Rdl* is located. To date, the *Rdl-A296S*/*T345S* allele pair has only been reported in Burkina Faso[61] which shares a border with northern Ghana and supports our finding of population connectivity across the northern arid regions of West Africa.

To investigate whether the different *Rdl* substitution pairs were associated with the chromosomal inversion karyotype on which the gene is located, we calculated the frequencies of *Rdl* substitutions for individuals with either the 2La or 2La + inversion. As expected, all individuals from the Upper East had the inverted 2La inversion karyotype and also the *A296S* and *T345S* substitution pair only found in this region (Fig. S5). Individuals from southern Ghana with the standard 2La inversion had the *A296G* and *T345M* substitution pair associated with the region, with substitution frequencies for 2La population cohorts ranging from 16–58%, but individuals with the inverted 2La inversion did not have either substitution pair. Interestingly, this was the case for individuals collected from the same cohort from Ashanti and the Central Region, suggesting an association of the *A296G* and *T345M* substitution with the 2La inversion karyotype.

Finally, we observed the *G280S* mutation and linked CNVs at the *Ace1* gene which are associated with resistance to organophosphates and carbamates[65, 66] (Fig. 5). These were present at > 5% in the population cohort from Greater Accra from 2012 only which formed a somewhat genetically differentiated group upon comparison with the other southern population cohorts on PCA analysis, including other southern populations sampled at the same time (Fig. 2A).

### Metabolic Resistance

**Figure 6:**
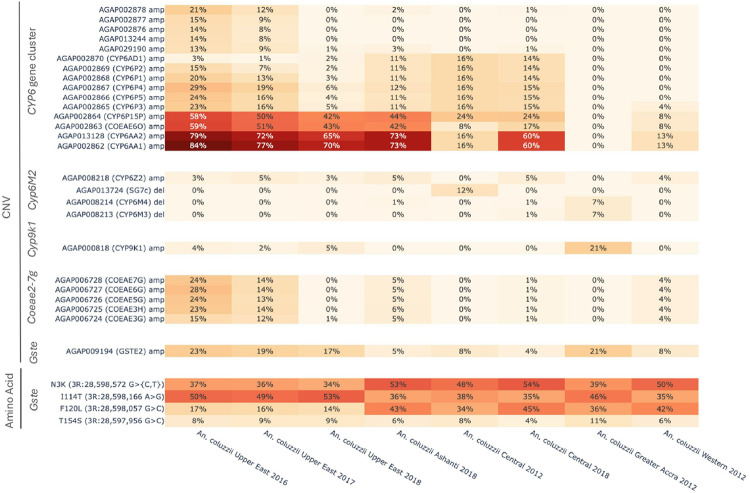
CNV and amino acid frequencies at metabolic insecticide resistance loci including the *Cyp6* gene cluster, *Cyp6m2, Cyp9k1*, the carboxylesterase gene cluster *Coeae2-7G* and *Gste*. Darker shades of red indicate higher frequencies.

To further investigate metabolic insecticide resistance in *An. coluzzii* across Ghana, we calculated the frequency of individuals within each population cohort with a copy number greater than two for genes associated with insecticide resistance, including cytochrome P450’s (AGAP002862-AGAP002870, AGAP000818, AGAP008212-AGAP008219), carboxylesterases (AGAP006228, AGAP006723-AGAP006728) and *Gste2* (AGAP009194). We also calculated amino acid substitution frequencies for the latter since the *Gste2*-*I114T* mutation is known to increase the activity of *Gste2*[36]. We found CNVs at the cytochrome P450 cluster *Cyp*6 on chromosome two (Fig. 6). Amplifications at the *Cyp6aa1* (AGAP002862) and 2 (AGAP013128) regions were present across Ghana at 60–84% frequency in the more recent population cohorts from 2018. This contrasts with the earlier sampling points from 2012, which presented frequencies between 0–16%, suggesting that metabolic resistance has risen in the country. In addition, we observed duplications at the carboxylesterase cluster *Coeae2g-7g*, which have been associated with resistance to pirimiphos methyl[35] at a higher frequency in northern Ghana (12–28%) than southern Ghana (0–6%), including cohorts collected in the same year (Fig. 6). *Gste2* CNV amplifications and the *I114* substitution were also present at higher frequencies in northern Ghana (17–23% and 49–53%, respectively) compared to southern Ghana (4–8% to 35–38%, respectively) (Fig. 6). The latter had similar *I114* frequencies across the different collection years. The exception was *An. coluzzii* from Greater Accra in 2012, which presented similar frequencies of both CNVs (21%) and the *Gste2*-*I114T* substitution (46%) to the more recently sampled northern populations. Moreover, we observed CNVs at the cytochrome *Cyp9k1* at 21% frequency in Greater Accra, while frequencies were ≤ 5% in all other population cohorts (Fig. 6), including southern cohorts collected at the same time. Overall, our findings indicate that metabolic resistance varies across Ghana, with the northern populations in general more impacted than the southern populations, although the region of Greater Accra had particularly high CNV frequencies at insecticide resistance loci.

### Signals of selection

**Figure 7:**
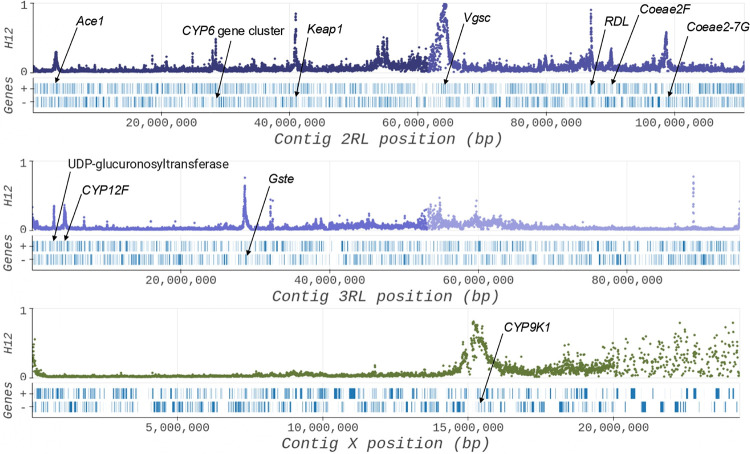
H12 selection scans for chromosomes 2RL, 3RL and X of the *An. coluzzii* population from the southern region of Greater Accra region in 2018. High values of H12 indicate a signal of positive selection. Signals at both known and novel loci putatively involved in insecticide resistance are annotated on the plot.

To identify signals of recent selection that may indicate novel insecticide resistance mechanisms, we calculated the H12 homozygosity statistic across windows of the genome and identified statistical peaks[67]. We observed a number of novel selection signals at loci which have not yet been functionally validated to confer insecticide resistance. We identified a selection peak over the *Keap1* locus (AGAP003645: 2R:40,926,195 − 40,945,169) in all cohorts from southern Ghana (Fig. 7 and Fig. S7). *Keap1* regulates the formation of the transcription factor Maf-S, known to trigger the expression of multiple metabolic resistance genes including cytochrome p450’s and glutathione S-transferases[68]. A selection signal has recently been observed at this gene in *An. arabiensis* from Kenya[69] and associated with deltamethrin treated survivors from Tanzania[35]. *Keap1* therefore provides a good candidate for functional validation. In addition, we observed selection signals unique to the cohort from Greater Accra (Fig. 7). We observed a selection signal close to the cytochrome *Cyp12f* (AGAP008019 3R:4,324,183-4,326,568) which is differentially expressed in permethrin and DDT resistant strains of *An. gambiae*[70]. Furthermore, a selection signal was present near a UDP glucuronosyltransferase (AGAP028055 3R:2,836,386-2,838,097) which is a class of uridine diphosphate (UDP)-glycosyltransferase (UGT) detoxification enzymes involved in xenobiotic metabolism[71]. Findings of selection signals over novel genes with a possible link to insecticide resistance in only Greater Accra in addition to our observation of high frequencies of known target-site and metabolic resistance mechanisms in this cohort, suggests that this region is under particularly high insecticide resistance pressure.

The selection signals we observed at known insecticide resistance loci agreed with the CNV and substitution frequencies generated for each population cohort. For example, we observed a selection peak at the *Cyp6* gene cluster (2R), *Vgsc* (2L) and *Gste2* (3R) for all population cohorts for which we also observed high frequencies of CNVs and resistance associated substitutions (Fig. S6–8). A signal at *Rdl* (2L) was apparent for the populations from the Upper East and Greater Accra, which both had relatively high frequencies of *Rdl* resistance associated substitutions. Additionally, Greater Accra had a signal at *Ace1* (2R) which was the only population cohort with CNVs and the G280S substitution at this locus. Interestingly, there was also a selection peak at *Coeae2f* and *Coeae2g-7g* on 2L for Greater Accra only although we only observed CNVs at appreciable frequencies in *An. coluzzii* from northern Ghana. It could be that the selection signal in Greater Accra is driven by another mechanism i.e., a substitution, although none have been identified as conferring resistance to date.

Although functional studies have not yet associated substitutions at *Keap1* with insecticide resistance, unique SNPs have been observed in haplotypes under selection for *An. arabiensis* from Kenya[69]. These included the substitution D780N and the stop gain mutation E762, which could result in loss of function and prevent the repression of detox gene expression in the absence of stress[46]. Therefore, we used diplotype clustering[34] to assess whether CNV duplications or SNPs were associated with haplotypes under selection in *An. coluzzii* from Ghana. We observed two haplotypes with low heterozygosity in southern Ghana for which we also observed a selection signal at the *Keap1* locus (Fig. S9). The two haplotypes were associated with a particular set of SNPs, including *V631I*, *V816F* and *V1001L* and *G788R* and *A943V*, respectively. In particular, the latter three SNPs are at higher frequencies in southern Ghana (43–52%) than northern Ghana (11–19%) and present at similar frequencies, suggesting they are either linked or have a synergistic effect (Fig. S10). These SNPs are different from those previously observed in East African *An. arabiensis*, but different molecular responses to insecticides are commonly observed across different taxa[61, 67]. The role of the variants we observed in insecticide resistance could be determined through validation studies and also provide a focus for longitudinal studies tracking the frequency and geographical spread of candidate variants.

We also used diplotype clustering to further investigate genomic variation at the Carboxylesterase cluster *Coeae2g-7g* since we observed a selection signal for *An. coluzzii* from Greater Accra despite the absence of CNVs. However, the dendrogram did not reveal any low heterozygosity clusters expected to result from selection on the region (Fig. S11). Increased sampling of the Greater Accra Region may improve resolution to detect selected variants.

## Discussion

Using whole genome sequence variation data from West African *An. coluzzii*, we observed population structure and divergent insecticide resistance mechanisms driven by gene flow, environmental variation, and differences in selection pressure from population control. We found two genetically different groups of *An. coluzzii* based on population structure analysis of a large number of SNPs and two chromosomal inversion regions, both with similar levels of nucleotide diversity. These two populations may represent *An. coluzzii* subject to distinct ecological selection pressures in the arid savannah region of northern West Africa and the wet deciduous forest region of the south. Isolation by distance could explain the pattern of population structure we have observed. However, since our sites in the south were ~ 500 km from those in the north and we did not have samples from intermediate sites, we were unable to determine whether this pattern reflects mosquito dispersal. However, the pattern of population structure between *An. coluzzii* from the different bioclimatic zones was present across West Africa where *An. coluzzii* are also expected to undergo long-range windborne dispersal to find suitable oviposition sites during the dry season[72]. Furthermore, there are no major geographical barriers expected to prevent mosquito dispersal across the investigated region. Therefore, another explanation for the population structure we observed is that reduced habitat suitability or local environmental adaptation restricts gene flow between northern (i.e Sahelian) and southern (ie. forest) West Africa. The former is supported by the geographical distribution of *An. coluzzii* which is bimodal because they predominate along the savannah and coastal regions[73], including in Ghana where aridity is increased in the north and southernmost coastal regions compared to the central zone[74]. However, it could also be that environmental adaptation leads to population specific mating or a fitness cost that restricts gene flow. Genomic evidence for environmental adaptation in *An. coluzzii* has been previously described based on the segregation of chromosome two inversions across West and Central Africa[59]. Similarly, we found that the inversion karyotypes of 2La and 2Rb segregated among *An. coluzzii* from northern and southern West Africa. Our findings support previous observations from Nigeria, Burkina Faso and Cameroon[12, 13, 15, 17] that the inverted 2La and 2Rb predominates in arid regions while standard karyotypes are common in humid forest environments. Such inversions have the potential to preserve locally selected alleles by reducing recombination between heterozygotes and are thought to be major mechanisms driving speciation and the formation of *Anopheles* ecotypes[3, 75]. Ecological studies comparing *An. coluzzii* across West Africa are lacking and therefore further work is required to determine whether distinct phenotypic and/or behavioral differences are observed between cohorts from the different bioclimatic zones. However, a preference for mating with the local population has been observed for *An. coluzzii* sourced from different ecozones in Burkina Faso under experimental conditions, which are analogous to those in other West African countries[9]. Distinct phenotypic differences have also been observed in summer dormancy, i.e., aestivation, from contrasting arid and mesic environments[11, 76]. Yet the question remains over whether aestivation in *An. coluzzii* has a genomic basis and can be linked to habitat use or geographical origin or whether it is a plastic response to dry season conditions, i.e., it is induced by the environment similar to the diapause response in other mosquitoes[76, 77]. It is also unknown to what extent aestivating *An. coluzzii* can increase population structure between cohorts. Overall, further experimental work linking ecologically driven phenotypes to genomic variation is required to untangle whether *An. coluzzi*i from contrasting environments in West Africa represent ecotypes adapted to different environmental conditions, but our findings raise this possibility.

The presence of ecologically distinct forms of *An. coluzzii* would have consequences for population control and malaria transmission since they will exhibit different life history traits and different responses to insecticide use. Indeed, we found that restricted gene flow between *An. coluzzii* across the different bioclimatic zones of northern and southern Ghana has led to different insecticide resistance profiles. Findings support previous work which found F_ST_ outlier regions at target site and metabolic resistance genes on comparison of forest and non-forest populations in Ghana, although comparisons did not include sites from northern Ghana[43]. Overall, we found that *An. coluzzii* from northern Ghana exhibited greater evidence for both target site and metabolic resistance than the southern populations, excepting the Greater Accra region. Along with higher frequencies of the known *Vgsc*-L995F substitution associated with pyrethroid resistance, northern Ghana also had the double substitution *Vgsc*-V402L and I1527T previously reported from nearby connected Burkina Faso[61]. Since this substitution pair provides pyrethroid resistance at a lower fitness cost than L995F[62], we expect it to rapidly spread across northern West Africa where we observed unrestricted gene flow. In addition, we observed higher frequencies of carboxylesterase CNVs and GSTE substitutions and CNVs in northern Ghana compared to the south, associated with multiple classes of insecticides including pirimiphos-methyl[40]. Although bed net use is widespread across the country, northern Ghana has a high malaria burden and has been additionally targeted with high coverage of long-lasting insecticide treated nets and IRS campaigns with propoxur between 2012 and 2016 [78, 79]. Despite a lower prevalence of molecular insecticide resistance, *An. coluzzii* from southern Ghana are still impacted mainly through target site resistance at *Vgsc*-L955F. In addition, we found that the populations in southern Ghana were uniquely impacted by a selection signal at *Keap1*. This gene is also under selection in East Africa and has the potential to impact the expression of detoxification genes to metabolise insecticides[46, 69]. For example, a reduction in the regulatory action of *Keap1* decreases mortality to organophosphates but increases mortality to pyrethroids and DDT[68]. Although it is unknown whether substitutions at the gene impact on insecticide resistance, we have identified several SNPs including V631I, V816F and V1001L and G788R and A943V which can be targeted for experimental work, including testing whether these variants have a synergistic effect. Although we also observed differences in *Rdl* substitutions between different bioclimatic zones and inversion karyotypes, the source of selection on this locus is unclear since dieldrin is no longer used in vector control. Possibly dieldrin use is ongoing, i.e., in agriculture, or the locus provides cross-resistance to another used insecticide.

We found that *An. coluzzii* from Greater Accra on the southern coast of Ghana were somewhat genetically divergent from other southern populations based on PCA and F_ST_ population differentiation statistics. In addition, they had unique molecular insecticide resistance mechanisms and novel selection signals on chromosome three. For example, we found appreciable frequencies of *Cyp9k1* CNVs, the *Ace1*-G280S substitution and *Ace1* CNVs at this locus in Greater Accra only, suggesting resistance to multiple classes of insecticides. In addition, we found novel signals of selection at the cytochrome *Cyp12f* linked to permethrin and DDT resistance in *An. gambiae*[70] and a UDP glucuronosyltransferase analogous to a UGT gene that confers resistance to the pyrethroid lambda-cyhalthrin and the neonicotinoid imidacloprid in fruitflys[71]. Findings suggest that *An. coluzzii* are under intense selection pressure from insecticides in Greater Accra. This notion is supported by bioassay data, which suggested particularly high resistance to pyrethroids and carbamates in *An. gambiae* from the region[80]. However, the findings of phenotypic resistance are not directly comparable with the genomic data since our sampling was from 2012 while the bioassay experiment was conducted in 2017. The findings are somewhat surprising given that malaria incidence and bed net use in Greater Accra is comparatively low to elsewhere in Ghana[81, 82]. However, Greater Accra is a populated urban area where there could be substantial use of household insecticide sprays for personal protection[83]. Furthermore, mosquito populations encounter increased pollution in urban environments, including habitats contaminated with insecticides from urban agriculture[73, 80]. For example, pollution from urban agriculture can result in similar levels of resistance when compared to populations from rural and cultivated areas[84–86]. Further study is required to identify the drivers of selection pressure within the city of Greater Accra. Even so, it is concerning that we have observed multiple and unique resistance mechanisms likely to reduce the efficacy of malaria control. Since we have found unrestricted gene flow across southern Ghana, any novel insecticide resistance mechanism has the potential to spread. However, this will also depend on selection pressure across the country, since resistance is often accompanied by a fitness cost[87, 88]. Experimental work through bioassays and/or association studies are required to confirm that the novel regions under selection confer a resistance mechanism and to pinpoint the genomic variation underlying the phenotype.

Our results demonstrate that gene flow among vector populations is important in influencing the distribution of insecticide resistance mechanisms. A full understanding of mosquito population structure, both regional and large-scale, is required to predict the success of gene drive technologies and how novel insecticide resistance mechanisms will spread when they arise in response to population control measures. This includes a greater understanding of how environmental conditions influence mosquito dispersal and connectivity, and how this may alter with climate change. A strong understanding of population connectivity is particularly important given the introduction of new technologies introduced to combat the rise in metabolic resistance, i.e., dual active ingredient (AI) nets. New technologies impose novel selection pressures and may be quickly challenged given *Anopheles* propensity for a rapid evolutionary response[61, 89, 90]. Any novel insecticide resistance mechanism will need to be quickly managed to maintain efficacy on their introduction. In tandem, large-scale routine monitoring of the temporal and geographical distribution of molecular insecticide resistance mechanisms across West Africa will be essential for a targeted defense.

## Supplementary Material

Supplementary Files

This is a list of supplementary files associated with this preprint. Click to download.

- Table1samplecollection.csv
- Table22La2Rbinversionkaryotypes.csv
- TableS1samplingtable.csv
- FigS5SupplementaryfigureRdlinversion.tif
- FigS9keap1diplotypeclustering.tif
- FigS10keapaa.tif
- FigS11Supplementaryfigurecoeaediplotypeclustering.tif
- FigS2Ghananjt.tif
- FigS3diversity.tif
- FigS7SignalsChr3.tif
- FigS1WestAfricanCountriesMap.tif
- FigS4Westafricanjt.tif
- FigS6SignalsChr2.tif
- FigS8signalsChrX.tif

## Figures and Tables

**Table 1:** The number of *An. coluzzii* collected from each sampling location in Ghana as part of the present study and details of other previously published datasets from West Africa included in analysis[40, 44, 58].

Country	Location	Latitude	Longitude	Year	No. samples	Study ID
Ghana	Upper East Region	10.85	−1.11	2018	563	This study
				2017	557	This study
				2016	204	This study
	Ashanti Region	6.18	−1.62	2018	274	Lucas *et al*. 2023
	Central Region	6.01	−1.87	2018	148	Lucas *et al*. 2023
		5.61	−1.55	2012	14	AG1000G Phase 3
	Eastern Region	6.09	−0.26	2012	1	AG1000G Phase 3
	Greater Accra Region	5.67	−0.22	2012	14	AG1000G Phase 3
	Western Region	4.91	−1.77	2012	24	AG1000G Phase 3
Burkina Faso	Hauts-Bassins	11.151	−4.235	2012	82	AG1000G Phase 3
				2014	53	AG1000G Phase 3
Mali	Koulikouro	12.68	−7.84	2004	11	AG1000G Phase 3
				2014	27	AG1000G Phase 3
	Segou	13.2	−6.13	2004	25	AG1000G Phase 3
	Sikasso	10.83	−7.81	2012	27	AG1000G Phase 3
Cote d’Ivoire	Lagunes	5.898	−4.823	2012	80	AG1000G Phase 3

**Table 2:** Percentage of 2La and 2Rb inversion karyotypes in population cohorts across the bioclimatic zones of West Africa using tagging SNPs for inversion karyotypes[19].

Country	Location	Year	Inversion karyotype %					
			2La+	2La+/2La	2La	2Rb+	2Rb+/2Rb	2Rb
**Cote d’Ivoire**	**Lagunes**	**2012**	41	45	14	93	8	0
**Ghana**	**Western Region**	**2012**	33	58	8	96	1	0
	**Central Region**	**2012**	48	36	16	100	3	0
		**2018**	34	50	16	98	3	0
	**Greater Accra Region**	**2012**	64	29	7	100	0	0
	**Eastern Region**	**2012**	100	0	0	100	0	0
	**Ashanti Region**	**2018**	46	45	9	96	10	0
	**Upper East**	**2016**	0	1	99	12	50	39
		**2017**	0	1	99	8	40	52
		**2018**	0	0	100	7	34	60
**Burkina Faso**	**Hauts-Bassins**	**2012**	0	2	98	46	41	12
		**2014**	0	4	96	42	40	19
**Mali**	**Koulikoro**	**2004**	0	0	100	9	73	18
		**2014**	0	4	96	4	33	63
	**Segou**	**2004**	0	4	96	8	32	60
	**Sikasso**	**2012**	0	4	96	30	59	11

## Data Availability

The sequences of the samples identified in this study were submitted to the European Nucleotide Archive (ENA) (Project: PRJEB2141, accessions ERR2656751-ERR9796298).
